# Perceived Stress and Psychological Impact Among Healthcare Workers at a Tertiaty Hospital in China During the COVID-19 Outbreak: The Moderating Role of Resilience and Social Support

**DOI:** 10.3389/fpsyt.2021.570971

**Published:** 2022-02-23

**Authors:** Qiaoyang Zhang, Guanzhong Dong, Weifen Meng, Zhuoyou Chen, Yin Cao, Min Zhang

**Affiliations:** The Affiliated Changzhou No. 2 People's Hospital of Nanjing Medical University, Changzhou, China

**Keywords:** 2019 novel coronavirus disease, healthcare workers, perceived stress, psychological distress, resilience, social support

## Abstract

**Aims:**

To investigate the psychological distress experienced by healthcare workers (HCWs) at a tertiary hospital in Changzhou, China, outside Wuhan, during the early stage of COVID-19 and evaluate the moderating effects of resilience and social support on the relationship between stress and psychological distress.

**Methods:**

The study was conducted between February 10 and 15, 2020, in a non-probabilistic way. The survey included questions regarding the risk of exposure, sociodemographics, perceived stress [10-item Perceived Stress Scale (PSS-10)], resilience [10-item Connor–Davidson Psychological Resilience (CD-RISC-10)], social support [Multidimensional Scale of Perceived Social Support (MSPSS)], and psychological distress [12-item General Health Questionnaire (GHQ-12)]. We applied the PROCESS macro for SPSS to test the hypotheses that resilience and social support moderated the stress response. In addition, a simple slope analysis was conducted when the interaction effect was statistically significant.

**Results:**

Some 33.6% of participants suffered from psychological distress (GHQ-12 ≥ 12). Perceived stress was positively related to psychological distress (*r* = 0.42, *p* < 0.001). In addition, resilience (ΔR^2^ = 0.03, *p* for interaction < 0.001) and social support (ΔR^2^ = 0.01, *p* for interaction <0.01) moderated the stress response. The impact of perceived stress on psychological distress was attenuated when subjects who were resilient (high β = 0.15, *p* < 0.001; low β = 0.36, *p* < 0.001), and perceived stress had less impact on psychological distress when social support was high (β = 0.24, *p* < 0.001) rather than low (β = 0.34, *p* < 0.001).

**Limitations:**

The cross-sectional design led to a lack of causal relationships between variables.

**Conclusions:**

Our data showed that resilience and social support moderated the stress response among HCWs in the pandemic, suggesting that improving resilience and social support could be appropriate targets to improve HCWs' mental health in the pandemic.

## Introduction

Wuhan, China, was hit with cases of life-threatening pneumonia caused by the novel coronavirus (COVID-19) in December 2019. Researchers found that the COVID-19 virus was more contagious than SARS, so COVID-19 quickly swept into every province in China. According to the Chinese government report, it had infected 58,016 people on February 17, 2020, and had caused more than 3,000 deaths within 2 months (1, 2). In addition, since healthcare workers (HCWs) have been the main force of the battle against the coronavirus, at least 1,716 HCWs had been infected with the virus by February 14, 2020, including six deaths (3).

A pandemic causes severe physical problems and different degrees of psychological distress (4, 5). While our hospital is located outside Wuhan, 30 cases have been confirmed in our city. As one of the biggest tertiary hospitals, our HCWs experienced an increasing workload and stress, especially when the knowledge about the disease transmission, disease course, and pathogenesis was limited during the first outbreak. Therefore, it has clinical significance to assess their mental health (5, 6). We applied the 12-item General Health Questionnaire (GHQ-12) to assess the severity of psychological distress among the HCWs in this hospital.

It is well known that different people react in different ways to stress. An individual's biological vulnerability, resilience, and social support could affect the psychological impact (7). As an ability to “bounce back”, resilience may strengthen stress resistance. Having a higher level of resilience leads to better mental health and decreased stress (8, 9). Individuals who receive social support typically get it from their family, friends, and other members of society. Social support can potentially improve coping with life stress and is highly associated with psychological status. We assumed that stress is related to psychological distress, and resilience and social support moderate their relationship.

In this study, we investigated the psychological distress among HCWs in our hospital when facing the first outbreak of COVID-19. In addition, we examined the effects of the moderators in the stress response, hoping to find effective buffers to avoid the psychological impact induced by the COVID-19 outbreak.

## Methods

### Sample Size

We applied the “Confidence Intervals for One Proportion” module of PASS software to calculate the sample size (10, 11). A sample size of 402 was calculated by a two-sided 95% confidence interval with a width equal to 0.1 when the sample proportion is 50%. The sample proportion is the estimated prevalence of psychological distress in our hospital based on other studies (12).

### Study Design

The questionnaire was uploaded to “Wenjuanxing” (www.wjx.cn), a secure survey website. To protect the privacy of the individuals, we kept the obtained data confidential and anonymous. The first page of the survey was an introduction of the study. Before conducting the survey, subjects were required to read and agree to the statement. Since the survey was open-ended, respondents were free to drop if they wished. All surveys with complete answers were allowed to be submitted. Depending on the Internet Protocol (IP) recorded by the online platform, a participant could submit only one time.

### Subjects and Procedure

The sample was recruited from February 10–15, 2020, and selected in a non-probabilistic way. We distributed the online survey link to staff *via* our hospital's official WeChat account. Besides, to increase the response rate, we encouraged the leadership of all departments to forward online questionnaire to all HCWs by WeChat groups. Finally, we obtained 1,098 responses from our electronic questionnaire. After removing 34 incomplete questionnaires, 1,064 individuals remained in our analyses.

The Clinical Research Ethics Committee of the Affiliated Changzhou No.2 People's Hospital of Nanjing Medical University approved the study.

### Measurements

Demographic information included age, gender, education, working occupation, chronic diseases, marital status, living with children, double staff, and major life events within half a year. Working occupations included doctors, nurses, technicians, and others (administrative and year-service staff). Regarding the risk of exposure, high risk was defined as working in the fever clinics, emergency department, suspected COVID-19 isolation units, and medical rescue team rushed to Wuhan.

Psychological distress was assessed by GHQ-12. The four-point Likert scoring method was applied in this study. Item responses range from 0 to 3, and the overall score ranges from 0 to 36, with more than 12 being defined as cases (12). The Chinese version has been validated in different groups of Chinese (13–15).

Perceived stress was measured by the 10-item Perceived Stress Scale (PSS-10). Participants are asked to report how they felt and thought during the last month. Each item ranges from 0 to 4, and overall score ranges from 0 to 40 (16). A large sample study showed that PSS-10 has good psychometric properties in Chinese populations (17).

Resilience was assessed by the 10-item Connor–Davidson Psychological Resilience (CD-RISC-10). Each item ranges from 0 to 4. The Chinese version has been validated (18, 19).

Social support was evaluated by the Multidimensional Scale of Perceived Social Support (MSPSS), a 12-item questionnaire. Each item ranges from 0 to 6. The Chinese version of the MSPSS has been shown to have excellent reliability and validity (20).

### Statistics

We applied SPSS 23.0 to conduct the analyses. Mean ± SD was used to describe the continuous variables. Categorical variables were expressed as frequencies (proportion). We used the independent-sample *t*-test and chi-square test to compare the non-case and case groups. Pearson's correlation analysis was applied in analyzing the relationship between key variables.

To test the hypotheses about moderating effect, we applied the PROCESS macro for SPSS, using the moderating Model 1 (21, 22), To determine if the moderation effect exists, the relationships for (i), (ii), and (iii) had to be significant—(i) direct effect of the predictor (perceived stress) on the outcome (psychological distress), (ii) direct effect of the moderator (social support or resilience) on psychological distress, and (iii) direct interactions effect (predictor × moderator) on psychological distress. PROCESS automatically calculates moderating effects and gives the change of R^2^ (ΔR^2^) due to interaction. If statistically significant, the slope analysis was carried out. The sociodemographic correlates were entered as control variables in the moderation model. *p* < 0.05 was statistically significant.

## Results

### Descriptive Analysis

As shown in Table 1, among the 1,064 valid questionnaires, 358 (33.6%) staff had symptoms of psychological distress. The rate of psychological distress was significantly higher in the high-risk exposure group than the low-risk group. Compared with the non-cases, the cases had a higher level of perceived stress but less resilience or social support (all *p* < 0.05). Besides, the proportion of chronic diseases, living with children, and major life events of cases were significantly higher than non-cases.

**Table 1:** Sociodemographic of the participants (*N* = 1,064).

	**Total (*n* = 1,064)**	**GHQ-12**		**t/χ^2^**	***p***
		**Non-case [*n* (%)]**	**Case [*n* (%)]**		
Risk of exposure, *n* (%)				17.198	0.000
High risk	345	199 (57.7)	146 (42.3)		
Low risk	719	507 (70.5)	212 (29.5)		
Age, years, mean ± SD	33.3 ± 8.2	32.9 ± 8.3	34.2 ± 8.1	−2.511	0.012
Gender, *n* (%)					
Male	180	127 (70.6)	53 (29.4)	1.714	0.191
Female	884	579 (65.5)	305 (34.5)		
Occupation, *n* (%)				9.725	0.021
Doctor	295	183 (62.0)	112 (38.0)		
Nurse	685	459 (67.0)	226 (33.0)		
Technician	10	5 (50.0)	5 (50.0)		
Other	74	59 (79.7)	15 (20.3)		
Education, *n* (%)				12.371	0.002
High school or less	17	6 (35.3)	11 (64.7)		
Undergraduate university or college	829	568 (68.5)	261 (31.5)		
Medical or graduate school	218	132 (60.6)	86 (39.4)		
Chronic diseases, *n* (%)				11.992	0.001
Yes	97	49 (50.5)	48 (49.5)		
No	967	657 (67.9)	310 (32.1)		
Marital status, *n* (%)				10.470	0.015
Unmarried	288	213 (74.0)	75 (26.0)		
Married	746	475 (63.7)	271 (36.3)		
Divorced	27	16 (59.3)	11 (40.7)		
Widowed	3	2 (66.7)	1 (33.3)		
Living with children, *n* (%)				15.105	0.000
Yes	672	417 (62.1)	255 (37.9)		
No	392	289 (73.7)	103 (26.3)		
Double staff, *n* (%)				2.463	0.117
Yes	238	168 (70.6)	70 (29.4)		
No	826	538 (65.1)	288 (34.9)		
Major life events, *n* (%)				16.147	0.000
Yes	125	63 (50.4)	62 (49.6)		
No	939	643 (68.5)	296 (31.5)		
PSS-10, mean ± SD	16.39 ± 5.65	14.98 ± 5.39	19.18 ± 5.08	−12.244	0.000
MSPSS, mean ± SD
Family supporting	22.35 ± 4.70	23.08 ± 4.42	20.89 ± 4.91	7.109	0.000
Friend supporting	21.49 ± 4.58	22.31 ± 4.30	19.87 ± 4.70	8.491	0.000
Other supporting	20.73 ± 4.63	21.65 ± 4.40	18.91 ± 4.53	9.506	0.000
CD-RISC-10, mean ± SD	27.31 ± 6.98	29.32 ± 6.62	23.34 ± 5.91	14.437	0.000

*GHQ-12, 12-item General Health Questionnaire; PSS-10, 10-item Perceived Stress Scale; MSPSS, Multidimensional Scale of Perceived Social Support; CD-RISC-10, 10-item Connor–Davidson Resilience Scale*.

### Correlation Analysis Among Key Variables

Table 2 shows intercorrelations among variables. Perceived stress was positively associated with psychological distress (*r* = 0.42, *p* < 0.001). Besides, psychological distress was negatively associated with resilience (*r* = −0.48, *p* < 0.001) and social support (*r* = −0.28, *p* < 0.001).

**Table 2:** Correlations between key variables.

	**1**	**2**	**3**
1 Perceived stress	–		
2 Resilience	−0.21***	–	
3 Social support	−0.09**	0.53***	–
4 Psychological distress	0.42***	−0.48***	−0.28***

** *p < 0.01*;

*** *p < 0.001*.

### Moderating Effect of Resilience

**Figure 1:**
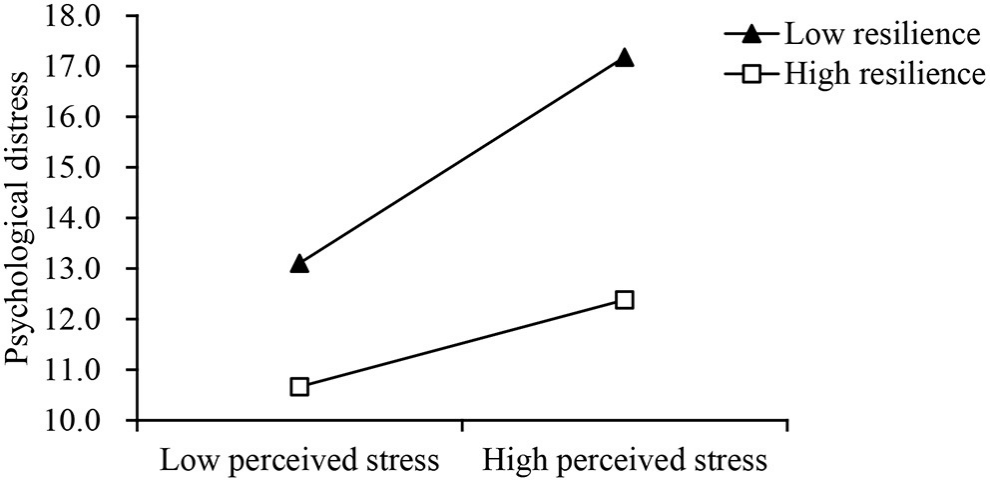
Interaction between perceived stress (x-axis) and low (−1 SD) and high (+1 SD) levels of resilience on psychological distress (y-axis).

As shown in Table 3, resilience (ΔR^2^ = 0.03, *p* for interaction <0.001) moderated in the relationship between perceived stress and psychological distress. Moreover, the impacts of perceived stress on psychological distress were attenuated when resilience was high (high β = 0.15, *p* < 0.001; low β = 0.36, *p* < 0.001). The interaction is visualized in Figure 1.

**Table 3:** Moderating effect of resilience.

**Effect^a^, variable**	**R^2^** **(*Δ*R^2^)**	***F***	**β**
The direct effect of perceived stress on psychological distress	0.07	12.27	0.31^***^
The direct effect of resilience on psychological distress	0.35 (0.28^***^)	71.63	−0.40^***^
The direct interactive effect (perceived stress × resilience) on psychological distress	0.38 (0.03^***^)	70.77	−0.16^***^

*** *p < 0.001*.

a *Adjusted for the sociodemographic*.

### Moderating Effect of Social Support

**Figure 2:**
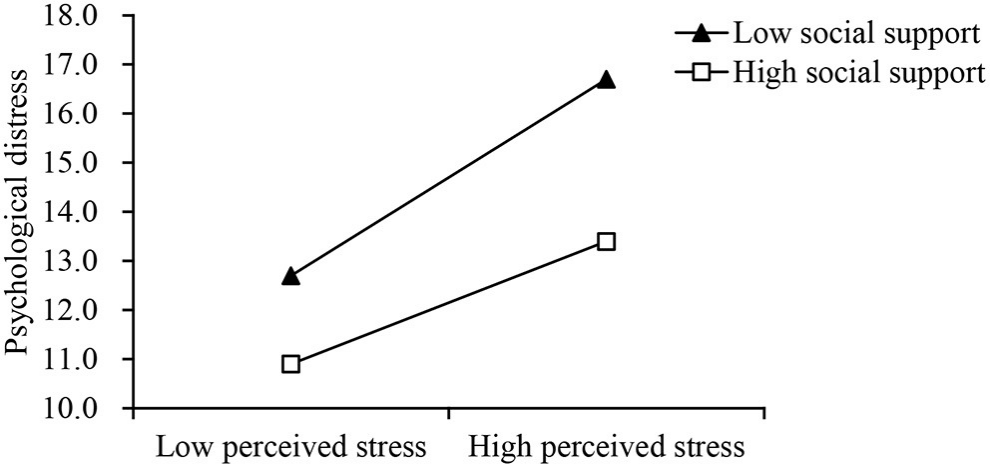
Interaction between perceived stress (x-axis) and low (−1 SD) and high (+1 SD) levels of social support on psychological distress (y-axis).

Table 4 shows that social support moderated in the relationship between perceived stress and psychological distress (ΔR^2^ = 0.01, *p* for interaction <0.01). As shown in Figure 2, perceived stress had less impacts on psychological distress when social support was high (β = 0.24, *p* < 0.001) rather than low (β = 0.34, *p* < 0.001).

**Table 4:** Moderating effect of social support.

**Effect^a^, variable**	**R^**2**^ (ΔR^**2**^)**	***F***	**β**
The direct effect of perceived stress on psychological distress	0.07	12.27	0.37^***^
The direct effect of social support on psychological distress	0.27 (0.19^***^)	45.45	−0.25^***^
The direct interactive effect (perceived stress × social support) on psychological distress	0.26 (0.01^**^)	41.84	−0.08^**^

** *p < 0.01*;

*** *p < 0.001*.

a *Adjusted for the sociodemographic*.

## Discussion

The present study found that 33.6% HCWs at this tertiary hospital experienced psychological distress during the first outbreak of COVID-19. In addition, the perceived stress was related to a higher psychological distress. Notably, resilience and social support moderated the stress response.

A mental health assessment of frontline HCWs is necessary to manage their stress and mental distress properly. A meta-analysis of studies conducted by April 17, 2020, found that the rate of depression and anxiety among HCWs in the pandemic was 23.2 and 22.8%, respectively (23). Notably, Feng et al. applied the GHQ-12 to examine the mental health of staff from 26 hospitals in Shanghai between February 9 and 21, 2020, and found that the rate of psychological distress was 47.7% (12). Considering Changzhou is relatively near to Shanghai (200 km), and both studies were conducted during the early stages of the pandemic, the result of our study was similar to what was found in Shanghai.

The present study also identified two moderators: resilience and social support, in the stress response among the HCWs during the outbreak of the COVID-19. Besides, data showed that the stress response was weakened with high resilience or social support. These findings were similar to other studies. A study in the Philippines reported that nurses with higher resilience and social support showed lower COVID-19 anxiety (24). Luceño-Moreno et al. found that resilience was the protective factor for Spanish HCWs' mental health (25). Notably, a large convenience sample study in the United States showed that HCWs who were more resilient had fewer COVID-19-related concerns, and higher resilience resulted in reduced anxiety and depression (26). Alnazly et al. reported significant correlations between social support and stress, anxiety, and depression among Jordanian HCWs in the pandemic (27).

During the early stage of the pandemic, HCWs may suffer from a decline in perceived support from friends or family due to the risk of infecting others and being isolated. Peer support appears to be necessary at this particular period, including support from colleagues and the hospital. A study in the Republic of Cyprus reported that organizational support was associated with HCWs' mental distress (28). In addition, another study in New York found that higher leadership support was linked to the lowest risk of depression, anxiety, and COVID-19-related post-traumatic symptoms (29). Therefore, the Columbia University Irving Medical Center program offers One-to-One Peer Support Sessions and Peer Support Groups to cope with psychological challenges in the pandemic (30). Besides, a hospital in France organized a safe place for HCWs to relax and support each other (31).

Meanwhile, building and maintaining resilience is essential for HCWs to recover quickly from the COVID-19 pandemic (32). The University of Minnesota Medical Center proposed a psychological resilience intervention to attenuate psychological distress for HCWs in the pandemic, founded on a system (Battle Buddies) developed by the American Army (33). In addition, researchers reviewed resilience strategies to cope with stress and mental health in the pandemic (34). They suggested several appropriate approaches, including mindfulness, resilience training, and staff feedback sessions. Notably, since the researchers successfully tested the effect of computer-assisted resilience training, intervention programs that apply this tool are needed to help HCWs.

## Limitation

This study has several potential limitations. The non-probabilistic sampling we used may somewhat hinder the validity of our findings, as has been reported in many published literatures during the early stage of the pandemic (26, 35, 36). However, our sample's prevalence of psychological distress was close to that of other studies conducted during the same period, indicating that our sample was somewhat representative of all HCWs in our hospital (12). In addition, the cross-sectional design led to a lack of causal relationships between variables. Moreover, residual confounding may exist because other factors could influence the outcome, such as income, coping skills, depression, anxiety, etc. Lastly, since this study was confined to a specific area (Changzhou), it will be necessary to conduct more studies to replicate these results.

## Conclusions

Overall, this study found that resilience and social support could moderate stress response among HCWs during the pandemic. To decrease the stress response in the pandemic, the psychosocial intervention for HCWs should pay much attention to the improvement of resilience and social support.

## Data Availability Statement

The raw data supporting the conclusions of this article will be made available by the authors, without undue reservation.

## Ethics Statement

The studies involving human participants were reviewed and approved by Institutional Review Board of Changzhou No.2 People's Hospital. The patients/participants provided their written informed consent to participate in this study.

## Author Contributions

MZ designed the study. QZ conducted the data analysis and wrote the manuscript. YC directed all the work. All authors contributed to the article and approved the submitted version.

## Funding

This work was funded by Application Foundation Program of Changzhou Science and Technology (CJ20180071).

## Conflict of Interest

The authors declare that the research was conducted in the absence of any commercial or financial relationships that could be construed as a potential conflict of interest.

## Publisher's Note

All claims expressed in this article are solely those of the authors and do not necessarily represent those of their affiliated organizations, or those of the publisher, the editors and the reviewers. Any product that may be evaluated in this article, or claim that may be made by its manufacturer, is not guaranteed or endorsed by the publisher.
